# Plasma Profiling of Acute Myeloid Leukemia With Fever‐ and Infection‐Related Complications During Chemotherapy‐Induced Neutropenia

**DOI:** 10.1002/cnr2.70024

**Published:** 2024-10-23

**Authors:** Iris C. Kreft, Annemarie van de Geer, Eva R. Smit, Carmen van der Zwaan, Floris P. J. van Alphen, Alexander B. Meijer, Erfan Nur, Arie J. Hoogendijk, Taco W. Kuijpers, Maartje van den Biggelaar

**Affiliations:** ^1^ Department of Molecular Hematology Sanquin Research Amsterdam The Netherlands; ^2^ Department of Blood Cell Research, Division Research and Landsteiner Laboratory of Amsterdam UMC Sanquin Blood Supply Amsterdam The Netherlands; ^3^ Department of Pediatric Immunology, Rheumatology and Infectious Diseases Emma Children's Hospital, Amsterdam UMC Amsterdam The Netherlands; ^4^ Department of Biomolecular Mass Spectrometry and Proteomics Utrecht Institute for Pharmaceutical Sciences (UIPS), Utrecht University Utrecht The Netherlands; ^5^ Department of Hematology Amsterdam UMC, location AMC Amsterdam The Netherlands

**Keywords:** acute myeloid leukemia, mass spectrometry, plasma, plasma profiling, proteomics

## Abstract

**Background:**

Acute myeloid leukemia (AML) is a heterogenous and complex blood cancer requiring aggressive treatment. Early identification and prediction of the complications following treatment is vital for effective disease management.

**Aims:**

We explored associations between plasma protein levels and fever‐ and infection‐related complications in 26 AML patients during chemotherapy‐induced neutropenia.

**Material and Methods:**

Longitudinal plasma profiling was conducted using data‐dependent mass spectrometry analysis.

**Results:**

Mass spectrometry‐based plasma profiling data correlated well with laboratory parameters, including C‐reactive protein, and revealed a broader inflammation protein network associated with fever‐ and infection‐related complications.

**Discussion and Conclusion:**

These data indicate the potential of longitudinal plasma profiling in AML patients for identifying and predicting complications that may aid in improved disease monitoring and treatment.

## 1 Introduction

Acute myeloid leukemia (AML) is the most common hematological malignancy with a complex underlying disease pathology [1]. Treatment of AML requires intensive chemotherapy that often results in neutropenia, characterized by an absolute neutrophil count below 0.5 × 10^9^/L [2] This renders patients highly susceptible to severe infectious diseases such as bacterial pneumonia and aspergillosis. Additionally, febrile neutropenia (FN), marked by fever in the context of neutropenia, poses a significant challenge in terms of prompt and accurate diagnosis of the causative pathogens [3]. Moreover, FN is associated with a mortality rate 4%–30% [4].

Therefore, accurate detection and prediction of complications in AML is crucial for optimizing treatment outcomes and avoiding undertreatment of patients. However, the heterogeneity among patients with FN poses challenges in accurately stratifying their risk for developing potentially severe complications during the episode. Various risk assessment models have been developed to classify patients at low and high risk for FN based on patient characteristics, including Talcott's Rule, the Multinational Association for Supportive Care in Cancer (MASCC) [5] and Modified Early Warning Signs (MEWS) [6] scores. In addition, protein biomarkers such as C‐reactive protein (CRP) [7] and procalcitonin (PCT) [8, 9] are commonly used in clinical practice for the prediction of fever complications (viral and bacterial infections) and death. However, their lack of specificity presents a challenge, as they are also elevated in various noninfectious conditions, including cancer itself and neutropenia [10, 11]. Therefore, other plasma components, including Interleukin‐8 (IL‐8) and nucleosomes, are currently being explored as potentially more selective biomarkers associated with infection‐related complications in AML patients undergoing chemotherapy [12].

In recent years, it has become clear that the plasma proteome can serve as indicator of changes in health status [13, 14, 15]. In clinical settings, longitudinal plasma profiling has been shown to be a particularly promising approach to identify molecular signatures associated with disease, including diabetes [16], multiple sclerosis [17] and corona virus disease‐2019 (COVID‐19) [18, 19, 20]. This study builds on previous published work by van de Geer et al. [12] and investigates whether plasma profiling can aid in identifying and predicting complications related to fever and infection in FN. Therefore, we performed longitudinal mass spectrometry (MS)‐based plasma profiling of 26 AML patients during chemotherapy‐induced neutropenia and correlated proteomics data with laboratory and clinical parameters to identify molecular signatures associated with fever‐ and infection‐related complications.

## 2 Materials and Methods

### 2.1 Study Cohort

In this study, patient samples from a prospective clinical study (NL54369.018.15) by van de Geer et al. [12] were used, including AML patients undergoing induction chemotherapy admitted to the Academic Medical Center location Amsterdam, The Netherlands (between Augustus 2016 and November 2018) (Table S1). Patients were included in the study based on a neutropenic episode (absolute neutrophil count < 0.5 × 10^9^/L) during their hospitalization. In total 4 relapsed and 22 newly diagnosed AML patients were included with varying risk categories (good, poor, very poor, and intermediate risk) and treatment protocols (Hemato‐Oncologie Volwassenen Nederland; Hovon) 103 A (*n* = 2) [21], Hovon 103 D (*n* = 2), Hovon 132 A (*n* = 15) [22], Hovon 132 B (*n* = 2) [22] for new diagnosed AML patients and Hovon 79 (*n* = 1) [23] and high‐dose cytarabine (*n* = 3) [24] and Hovon 103 A (*n* = 1) [21] for relapsed patients. The study was conducted according to the Declaration of Helsinki and was approved by the Medical Ethics Committee of the Amsterdam UMC, Amsterdam, The Netherlands. Written informed consent was obtained from all study participants. The distribution included 13 patients with one neutropenic episode, 11 patients with two episodes, and 2 patients with three episodes (Table S2). Neutropenic episodes were categorized into three groups: (1) non‐FN defined as non‐fever, (2) mild‐FN defined by the presence of fever and absence of infection‐related complications, and (3) complicated‐FN defined as a FN episode with a severe course and requirement for ICU admission and/or death (Figure S1A).

### 2.2 Blood Collection and Conventional Laboratory Tests

Blood sampling was performed prospectively upon the onset of neutropenia (absolute neutrophil count < 0.5 × 10^9^/L). Sampling occurred 2–3 times weekly throughout the entire episode, with increased frequency during fever. Sampling was stopped when neutrophil counts raised > 0.5 × 10^9^/L. Blood was obtained by venipuncture using the vacutainer system (Becton Dickinson, Plymouth, UK) at Amsterdam UMC, location AMC. Plasma was obtained by whole blood centrifugation at 100*g* for 15 min at room temperature (RT) followed by centrifugation of platelet rich plasma (PRP) at 1000*g* for 15 min and aliquots were stored at −80°C until analysis. Separate aliquots of plasma were transferred to Sanquin Research, Amsterdam, The Netherlands. Plasma CRP, interleukin 8 (IL‐8), human neutrophil elastase (HNE), myeloid‐related protein‐8/14 (MRP8/14) and nucleosomes levels were determined by ELISA, as described previously [12] and plasma profiling was performed as described below.

### 2.3 Sample Preparation for Proteomic Analysis

For proteomic analysis, a fresh aliquot from each plasma sample was used and all samples were processed in a single batch (February 2020) (Figure S1B). Plasma was diluted 1:20 with 100 mM tris[hydroxymethyl]aminomethane (Tris) buffer (pH = 8.0) and 2.86 μL plasma was mixed with 10 μL 1% vol/vol sodium deoxy cholate (BioWORLD, USA), 10 mM tris(2‐carboxyethyl)phosphine (TCEP) (Thermo Fisher Scientific, USA), 40 mM chloroacetamide (CAA) (Sigma–Aldrich, Germany) in 100 mM Tris buffer (pH = 8.0) (Life Technologies, UK). After incubation at 95°C for 5 min, samples were cooled down to RT and proteins were digested overnight at 25°C with MS‐grade trypsin Gold (Promega, Madison, WI, USA) (in a protein:trypsin ratio of 100:1). The enzymatic reaction was quenched with 100 μL of 1% (vol/vol) trifluoroacetic acid (TFA; Thermo Fisher Scientific, USA). Samples were desalted by solid phase extraction (SPE) using C18 cartridges on the AssayMAP Bravo platform according to manufacturer's protocol (Agilent Technologies, USA). The eluate was completely dried using a SpeedVac centrifuge at 45°C (Eppendorf Concentrator plus), resuspended in 12 μL 2% vol/vol acetonitrile and 0.1% vol/vol TFA.

### 2.4 Mass Spectrometry Analysis

Samples containing ±500 ng tryptic peptides were separated on a C18 column installed on a Dionex Ultimate 3000 RSLC nano LC System coupled to a Nanospray Flex Ion Source and Orbitrap Fusion Lumos Tribrid MS (Thermo Fisher Scientific) operating in data‐dependent (DDA) mode. Peptides were loaded on a 20 cm fused‐silica emitter (75 μm inner diameter, 360 μm outer diameter) (New Objectives, USA) packed in‐house with ReproSil‐Pur C18‐AQ, 1.9 μm resin (Dr Maisch GmbH, Germany). The column was installed on a Dionex Ultimate3000 RSLC nanoSystem (Thermo Fisher Scientific) using a titanium Tee union formatted for 360‐μm outer diameter columns (IDEX) and a liquid junction. The column temperature was kept at 40°C by a PRSO‐V2 column oven (Sonation Lab solutions). The spray voltage was set to 2.15 kV. Buffer A was composed of 0.1% formic acid and buffer B of 0.1% formic acid, and 80% acetonitrile. Peptides were loaded for 17 min at 300 nL/min at 5% buffer B, equilibrated for 5 min at 5% buffer B (17–22 min), and eluted by increasing buffer B from 5% to 27.5% (22–122 min) and 27.5%–40% (122–132 min), followed by a 5‐min wash to 95% and a 6‐min regeneration to 5%. Survey scans of peptide precursors from 375 to 1500 *m*/*z* were performed at target. Tandem MS (MS/MS) was performed by isolation with the 120 K resolution (at 200 *m*/*z*) with a 4 × 10^5^ ion count quadrupole with isolation window 0.7, HCD fragmentation with a normalized collision energy of 30, and rapid scan MS analysis in the ion trap. The MS/MS ion count target was set to 3 × 10^4^ and the max injection time was 20 ms. Only those precursors with charge state 2–7 were sampled for MS2. The dynamic exclusion duration was set to 30 s with a 10‐ppm tolerance around the selected precursor and its isotopes. Monoisotopic precursor selection was turned on. The instrument was run in top speed mode with 3‐s cycles.

### 2.5 Data Analysis

RAW files were acquired with Xcalibur software 4.1 (Thermo Fisher Scientific, USA) and processed with the MaxQuant (MQ) 1.6.2.10 software [25]. Peptides were [25] searched against the Homo Sapiens Uniprot database (accessed February 2019), using standard MQ settings and the “match between runs” option enabled. Protein output files were processed in RStudio (version 4.03) and proteins were filtered based on “reverse” and “only identified by site” criteria. Label free quantitation (LFQ)‐intensities were log_2_‐transformed and only proteins present in at least 75% of all samples were considered for quantification, which equals 165 out of the 219 measured samples (Table S4). Missing values were imputed by drawing random samples from a normal distribution with a mean shifted downward by 1.8 standard deviation and a standard deviation scaled (width) to 0.3 relative to the abundance distribution of the corresponding proteins across all samples in the data set (Table S5). This imputation resulted in an average of six proteins per sample, accounting for 2.26% of the total proteins quantified.

### 2.6 Bioinformatics Analysis

Bioinformatic analyses were performed in R (Figure S1C). For intra‐individual protein variation analysis, we used non‐log‐transformed (RAW) protein intensities (*n* = 225) to compute the coefficient of variation (CV) per patient for each quantified protein. Next, we determined the log fold change difference in protein level between the first time point and the average of subsequent time points (e.g., time point 2, 3, 4, 5, 6, etc.). To determine the protein level variation within the entire cohort, we calculated the median CV per patient for each of the 225 proteins quantified as well as the adjusted log‐fold change. The adjusted log‐fold change forces all patients to have the same mean intensity value for each measurement, by subtracting the median LFQ‐intensity of each patient from the overall average LFQ‐intensity [26].

For correlation analysis, Spearman correlation was calculated between all quantified proteins (*n* = 225) and CRP, IL‐8, MRP8‐14, HNE, and nucleosome levels and the FN‐category (non‐fever, mild‐FN, complicated‐FN), binary category fever (presence or absence of fever at the time of sampling) and fever‐cause categories (including unknown cause, bacterial‐, fungal‐, multifactorial, or non‐infectious). Only Spearman correlation coefficient > 0.4 were constructed in a network, supplemented with proteins that also correlate > 0.4 to those identified in the global correlation analysis.

For statistical analysis of significantly altered proteins between fever and non‐fever samples, we used a one‐sample *t*‐test with a false discovery rate of < 0.05 after Benjamini‐Hochberg correction using the limma'‐package [26].

### 2.7 Data Availability

All raw MS files and search/identification files obtained with Maxquant have been deposited in the ProteomeXchange consortium via the PRIDE partner [27] repository with identifier PXD040526. Additionally, we have made the data used for bioinformatics analysis available in a readily accessible and searchable format for the benefit of the scientific community (Tables [Link], [Link], [Link], [Link], [Link], [Link]).

## 3 Results

### 3.1 Plasma Proteome Dynamics in AML Patients

**FIGURE 1:**
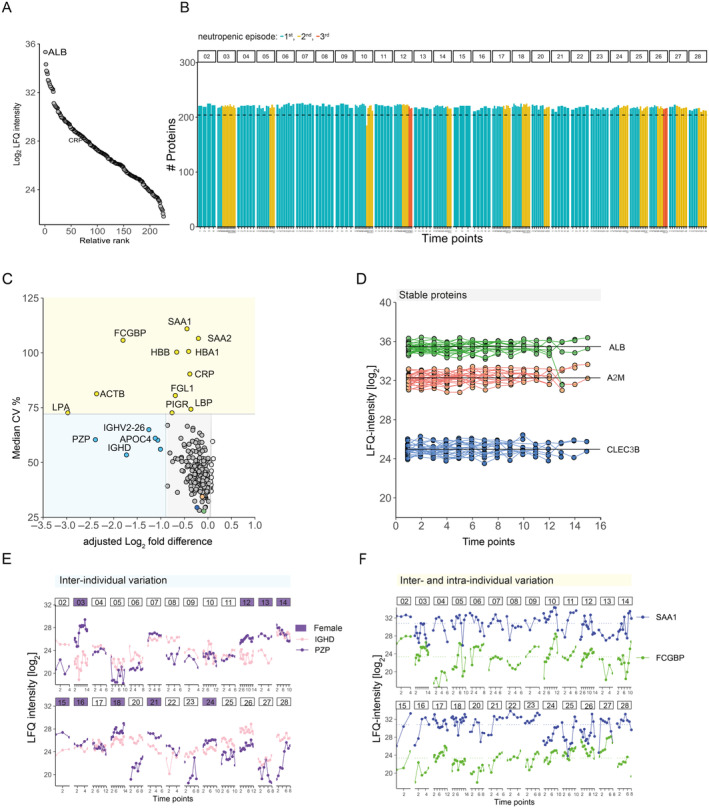
Plasma protein dynamics variation in AML patients. (A) The median quantitative label‐free intensity (LFQ) values of 225 plasma proteins ranked by their abundance. (B) The number of quantified proteins in each sample is categorized by patient at the top and color‐coded based on the neutropenic episode: First episode (blue bars), second episode (yellow bars), and third episode (red bars). (C) Average of coefficient of variation (mean CV%) in global plasma proteome quantification in relation to the adjusted log_2_ fold distance from all patients. Proteins with high inter‐ and intra‐individual variation of CV > 75% were annotated in yellow. Proteins with higher inter individual variation of a CV < 70% and adjusted log_2_ fold difference were annotated in blue. Proteins with stable levels falling within the 95th percentile were annotated in grey. (C) LFQ‐intensities levels of relatively stable proteins were plotted over time points for all individuals, albumin (ALB, green), alpha 2 macroglobulin (A2M, red), and c‐type lectin domain family 3 member B (CLEC3B, blue). (D) Distribution of LFQ‐intensity levels of proteins with high inter‐individual variation, immunoglobulin heavy constant delta (IGHD, pink), and pregnancy zone protein (PZP, purple) categorized per patient. Purple boxes denote female subjects. (E) Distribution of LFQ‐intensity levels of proteins with high inter‐and intra‐individual variation, serum amyloid A1 (SAA1, blue) and Fc gamma binding protein (FCGBP, green) categorized per patient.

To draw a detailed picture of the dynamic nature of circulating plasma proteins in response to chemotherapy‐induced neutropenia with fever and infection‐related complications, we investigated longitudinal blood plasma samples of 26 AML patients using MS‐based proteomics, including a total of 219 samples with an average of 8 time points (range 4–14 time points) per individual over an average period of 33 sampling days (15–52 days). Across all 219 samples, we quantified a total of 225 proteins over a large dynamic range (Figure 1A). The median number of quantified proteins in samples from AML patients was 219 (±4), indicating that this method is reliable and robust across all samples (Figure 1B).

First, we assessed the protein variation across individuals, we plotted the longitudinal protein CVs for each patient against the log_2_ fold‐difference. This analysis showed a considerable variation in the plasma proteome in the majority of the patients, which included both individual‐specific signatures (e.g., coagulation factor XIII A chain [F13A] in patient (BIOM)26) as well as common signatures across multiple patients (Figure S2 and Table S6). To visualize the average variation across the entire patient cohort, we plotted the mean CVs against the adjusted log_2_ fold‐difference. The majority of proteins (*n* = 204) exhibited relatively stable abundances as indicated by the grey box, which corresponds to the 95th percentile of the log_2_ fold‐difference and a CV < 70% (Figure 1C). Stable proteins included albumin, alpha‐2‐Macroglobulin (A2M) and c‐type lectin domain family 3 member B (CLEC3B) (Figure 1D). Proteins with substantial inter‐individual variability, defined by a CV < 70% and a log_2_ fold‐difference outside of the 95th percentile, included immunoglobulin heavy constant delta (IGHD), apolipoprotein C4 (APOC4), immunoglobulin heavy variable 2–26 (IGHV2‐26) and pregnancy zone protein (PZP). Notably, PZP levels were higher abundant in females compared to males (Figure 1E). Furthermore, proteins with high inter‐ and inter‐variation defined by a mean CV > 75%, included hemoglobin subunits (HBA1, HBB), and acute phase proteins such as serum amyloid A1/A2 (SAA1, SAA2), CRP, lipopolysaccharide‐binding protein (LBP) as well as Fc Gamma binding protein (FCGBP), polymeric immunoglobulin receptor (PIGR) and fibrinogen‐like protein 1 (FGL‐1) (Figure 1F).

### 3.2 Correlation of Plasma Proteins With Fever‐ and Infection‐Related Complications in AML

**FIGURE 2:**
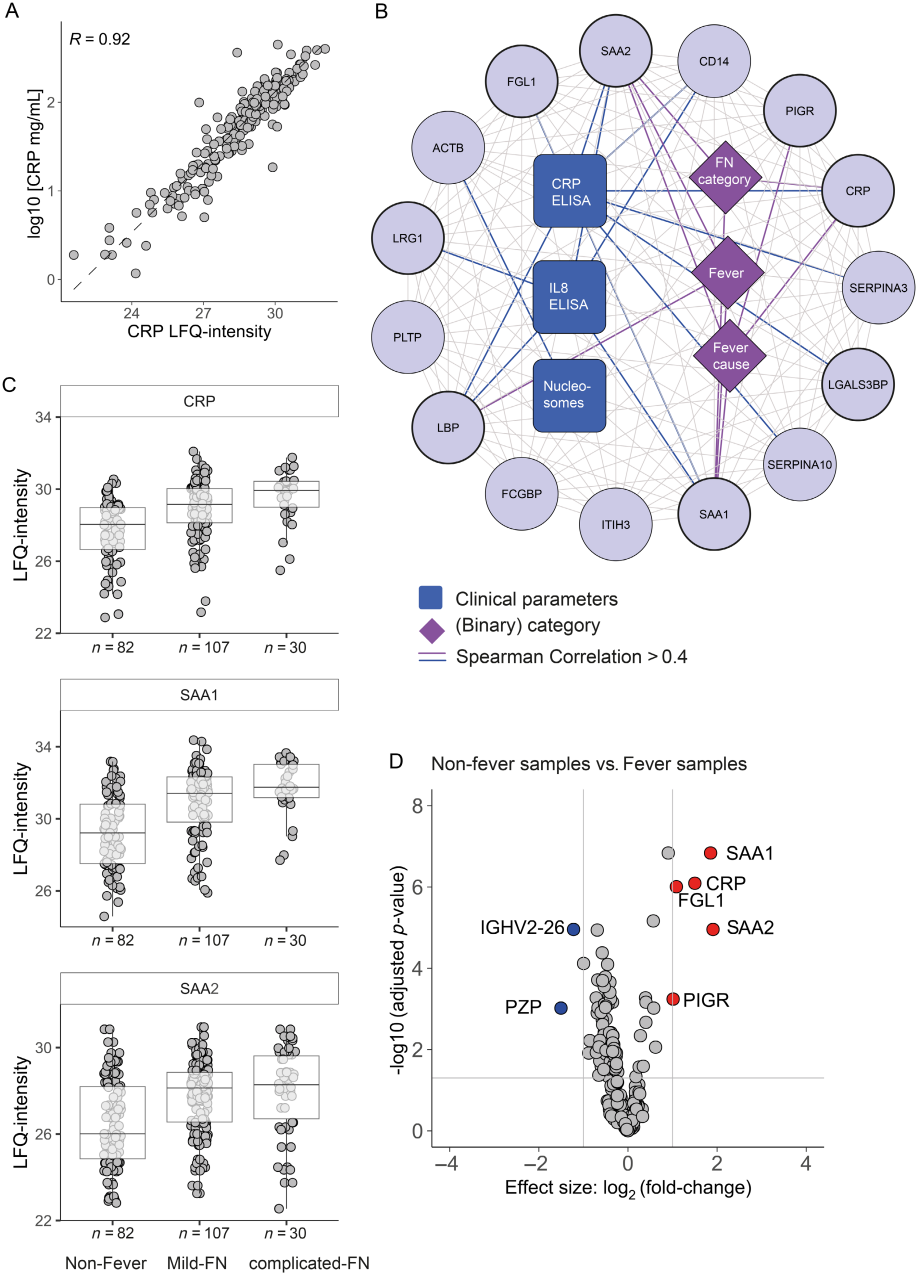
Plasma inflammation protein network associated with fever and infection‐related complications. (A) CRP levels quantified by enzyme‐linked immunosorbent assay (ELISA) (*x*‐axis) and mass spectrometry (*y*‐axis) strongly correlates (*R* ^2^ = 0.92). (B) Global network representing the connection between inflammatory related proteins (circles), clinical parameters (squares), and binary groups including FN categories, fever subtypes, and fever causes (diamonds). Only correlation coefficients > 0.4 are shown with bold lines the correlations between proteins and clinical and binary categories. (C) Boxplots depicting LFQ‐intensities of serum amyloid A1 (SAA1), C‐reactive proteins (CRP), and polymeric immunoglobulin receptor (PIGR) plotted for each (FN) category (non‐fever, mild‐FN, complicated‐FN). (D) Comparison of the patients samples with fever and non‐fever. Volcano plot with *x*‐axis depicting the protein level fold change and the *y*‐axis the −log10 *t*‐test *p*‐values (adjusted Benjamini Hochberg *p* < 0.05 and effect size |logFC| > 1). Proteins with significant increased levels in fever samples are annotated in red and proteins with decreased abundance in blue.

To assess the agreement of our proteomic data with clinical laboratory data, we compared the levels of CRP quantified by ELISA and MS which resulted in a high correlation (*R* = 0.92, *p*‐value = 2.2 × 10^16^) (Figure 2A and Table S7). Prompted by this observation, we correlated all our proteomics data with available laboratory parameters, including ELISA‐based levels of IL‐8, HNE, myeloid‐related protein‐8/14 and nucleosomes as well as the FN‐category (non‐fever, mild‐FN, complicated‐FN), binary category fever (presence or absence of fever at the time of sampling) and fever‐cause categories (including bacterial‐, fungal‐ or multifactorial‐infection, noninfectious or unknown cause). This analysis revealed an extended network of 15 proteins, including actin (ACTB), CRP, FGL1, galectin‐3‐binding protein (LGALS3BP), ITIH3, LBP, LRG1, monocyte differentiation antigen CD14 (CD14), phospholipid transfer protein (PLTP), PIGR, SAA1, SAA2, serpin family A member 3 (SERPINA3), and serpin family A member 10 (SERPINA10) (Figure S3 and Figure 2B). The strongest correlations were observed between CRP ELISA levels and LFQ values of SAA1 (*R* = 0.86), SAA2 (*R* = 0.81), LBP (*R* = 0.78), FGL1 (*R* = 0.64), and LRG‐1 (leucine‐rich alpha‐1‐glycoprotein, *R* = 0.63). In addition, IL‐8 ELISA levels correlated with a subset of these proteins, including SAA1 (*R* = 0.55), SAA2 (*R* = 0.5), and LBP (*R* = 0.48). Furthermore, the FN‐category correlated with inflammation‐related proteins such as CRP (*R* = 0.44), SAA1 (*R* = 0.44), and SAA2 (*R* = 0.40), and their levels were indeed increased in samples obtained from patients with mild‐FN and complicated‐FN compared to non‐fever patient samples (Figure 2C). We also examined the molecular signatures associated with fever by comparison of the plasma proteomes in absence or presence of fever. LFQ levels of five proteins (CRP, SAA1, SAA2, PIGR, and FGL1) were significantly more abundant and levels of PZP and immunoglobulin heavy chain variable region 26 (IGHV‐26) were significantly decreased in the fever sample group compared with the non‐fever sample group, which is consistent with the correlation analysis (Figure 2D and Table S8). Furthermore, we found that fever‐cause categories showed the strongest correlation with PIGR (*R* = 0.47), SAA1 (*R* = 0.46), and SAA2 (*R* = 0.42), although no distinct pattern was discernible for fever‐cause. Overall, our co‐expression analysis demonstrated a strong correlation between a network of inflammatory plasma proteins and fever‐related parameters (Table S3), suggesting a potential role for these proteins in AML patient disease monitoring.

## 4 Discussion

Identification and prediction of infections and infection‐related complications in treatment of AML patients is of great importance for effective disease management. Here, we conducted a plasma‐based proteomics approach to profile longitudinal plasma samples from 26 AML patients during chemotherapy‐induced neutropenia with fever‐ and infection‐related complications. We demonstrate that while a large proportion of proteins in plasma remained relatively stable between and within individuals, a subset of plasma proteins showed a high inter‐ and intra‐individual variation, indicating heterogeneity among and within patients. The main drivers of this variation in plasma protein levels were inflammation‐related proteins, as well as hemoglobin subunits. The contribution of hemoglobin subunits herein was likely due to sample handling [28] or as a result of low blood cell counts after chemotherapy [12]. Furthermore, our findings indicate sex‐specific alterations in PZP, consistent with previous findings [13, 14].

Co‐expression analysis revealed an association of levels of inflammation and immune response proteins, including CRP, SAA1, SAA2, PIGR, FGL1, LBP, LRG1, and LGALS3BP, with fever category and FN‐categories. Among these proteins, CRP, SAA1, and SAA2 are described as common markers for inflammation [13, 29] and combined monitoring of CRP and serum amyloid A has previously been shown to increase specificity for monitoring viral infections [30, 31, 32]. The observed correlation with additional proteins suggests that monitoring a broader panel of inflammatory‐ and immune‐related proteins may have additional merit. Of these proteins LGALS3BP has been previously described to have both prognostic and functional roles in cancer [33] and has also been described to be elevated together with PIGR and LBP in infectious diseases like COVID‐19 [34, 35]. FGL1 functions in regulating immune response and has been suggested as a therapeutic target in inflammatory conditions [36]. In addition, the multi‐functional secretory glycoprotein LRG1, has recently emerged as a potential biomarker in cancer patients at high risk for disease progression and recurrence [37]. Furthermore, in our correlation network, we identified CD14, which plays a crucial role in recognizing bacterial lipopolysaccharide (LPS), thereby triggering immune responses [38]. Recently, Presepsin (PSEP), also known as soluble CD14, has demonstrated potential as a prognostic biomarker for severe bacterial infections [39].

To our knowledge, this is the first study that evaluates plasma profiles using MS‐based proteomics in AML patients with FN. The simultaneous detection of multiple proteins offers advantages over traditional immunoassays like ELISA, which monitors only single proteins. A specific strength of our study is the unbiased nature of our approach, which enables a comprehensive analysis of the plasma proteome of AML patients with chemo‐induced neutropenia and discovery of proteins linked to fever‐ and infection‐related complications. Other omics technologies have been applied, including metabolomics and transcriptomes to investigate FN in patients with hematological malignancies [40, 41, 42, 43]. Lappalainen et al. utilized nontargeted metabolomics and found potential novel biomarkers for FN, including an androgen hormone, citrulline, and phosphatidylethanolamine that also correlated with CRP and PCT. While some studies have utilized MS‐based proteomics to identify protein biomarkers for stratifying and predicting mortality in septic patients, this approach has not been applied to patients with FN [44, 45]. Moreover, our analysis included longitudinal samples from AML patients which allows for the correction of inter‐individual variations and tracking of changes over time, offering a more accurate understanding of disease progression. Taken together, one could speculate that, while blood culture techniques are the gold standard for identifying bacterial and fungal infections [46], plasma‐based protein biomarker panels may be a better and faster indication of the infection status in patients with febrile symptoms.

Several limitations of our study need to be addressed. First, the study was performed in a modest number of patients with unequally distributed patient categories, such as fever cause, number of neutropenic episodes, occurrences of relapse, and diverse chemotherapy regimens. Furthermore, our study was conducted prospectively and sampling was stopped when patients were no longer neutropenic. As a result, there were discrepancies in sampling frequency and the number of samples collected from each patient, which may introduce biases in the analysis. Furthermore, no healthy controls were included in the study, which limits the ability to compare patient data to a baseline of healthy protein expression. Lastly, the depth of our proteomic analysis was limited, hindering the detection of low‐abundant proteins. This issue could be addressed by applying depletion strategies or utilizing different acquisition methods like data‐independent acquisition or targeted approaches to enable the identification and quantification of low‐abundant proteins [47], such as interleukins, nucleosomes, and HNE. Alternatively, affinity‐based proteomic assays could be employed, such as the Olink 96 immuno‐response protein panel which includes cytokines, cytokine receptors, members of the chemokine ligands and pro‐apoptotic proteins [48].

In conclusion, our study highlights the potential of longitudinal plasma profiling in AML patients. Although the actual predictive value of these proteome patterns needs to be studied prospectively, these findings suggest a major role for inflammatory‐related proteins in development and individual prediction of fever‐ and infection‐related complications in AML patients, as well as their potential relevance in other hematological malignancies or pathogen‐induced diseases. To further assess the clinical diagnostic utility of the above‐mentioned proteins, the next step should involve conducting validation studies in larger patient cohorts, performing receiver operator characteristic (ROC) analysis and implementing randomized control trials. With ongoing improvements in technology leading to increased proteomic depth and throughput, we envision that unbiased discovery proteomics could play a beneficial role in advancing prognosis and prediction of complications in cancer patients. Additionally, it may provide valuable insights into the underlying pathophysiology of AML and treatment responses in future studies.

## Author Contributions

**Iris C. Kreft:** conceptualization (lead), data curation (lead), formal analysis (lead), investigation (lead), methodology (equal), validation (lead), visualization (lead), writing – original draft (lead), writing – review and editing (lead). **Annemarie van de Geer:** conceptualization (equal), writing – review and editing (equal). **Eva R. Smit:** data curation (equal), formal analysis (equal), writing – review and editing (equal). **Carmen van der Zwaan:** formal analysis (equal), methodology (equal), writing – review and editing (equal). **Floris P. J. van Alphen:** formal analysis (supporting), methodology (supporting), writing – review and editing (equal). **Alexander B. Meijer:** funding acquisition (supporting), writing – review and editing (equal). **Erfan Nur:** resources (supporting), writing – review and editing (supporting). **Arie J. Hoogendijk:** investigation (equal), methodology (equal), software (supporting), supervision (supporting), writing – review and editing (equal). **Taco W. Kuijpers:** formal analysis (supporting), supervision (supporting), writing – review and editing (equal). **Maartje van den Biggelaar:** conceptualization (equal), funding acquisition (lead), project administration (lead), supervision (lead), writing – original draft (lead), writing – review and editing (lead).

## Conflicts of Interest

The authors declare no conflicts of interest.

## Supporting information


**Figure S1:** Study design (A) Patient Cohort: 26 AML patients with chemo‐induced neutropenia underwent longitudinal blood sampling (neutrophil count < 0.5 × 10^9/L). Samples categorized by FN status: non‐fever (triangles), mild‐FN (dots), complicated‐FN (squares). (B) Proteomic Setup and Analysis:Plasma proteins were digested into peptides and analyzed via label‐free LC–MS/MS. Data processed using MaxQuant and Rstudio. (C) Bioinformatic Analysis Flow Chart: MaxQuant analysis produced protein output files. All steps of data analysis corresponded with the figures.
**Figure S2:** Longitudinal protein‐specific coefficients of variation (CVs) calculated per patient (BIOM) vs. the log‐2‐fold difference from first time point of each individual and the average of the subsequent time points.
**Figure S3:** LFQ‐intensities of four proteins that correlated with fever cause from the Spearman correlation analysis. The levels C‐reactive protein (CRP), polymeric immunoglobulin receptor (PIGR), serum amyloid A1 (SAA1), and serum amyloid A2 (SAA2) are plotted across different fever causes, bacterial infection, fungal infection, multifactorial, non‐infectious, unknown focus.

**Table S1:** Patient characteristics and details on the febrile neutropenic episodes adapted from van de Geer et al. [6].

**Table S2:** Characteristics of patients, including the total number of samples analyzed, the neutropenic episodes experienced, and the number of samples per neutropenic episode.

**Table S3:** Overview of Study Cohort and Analytical Procedures Described in the Methods Section.

**Table S4:** Number of proteins quantified by LFQ intensity levels in this study, categorized by patient number and time points.

**Table S5:** Number of proteins quantified by imputed LFQ intensity levels in this study, categorized by patient number and time points.

**Table S6:** Coefficient of variation versus Log fold change from time point 1 against the average of subsequent time points.

**Table S7:** Spearman correlation coefficients derived from the correlation between proteins quantified, clinical data and binary category.

**Table S8:** Significantly expressed proteins resulting from the comparison of fever and non‐fever samples, utilized in Figure 2D.

**Table S9:** A list of proteins that exhibit correlations with clinical and laboratory data in correlation analysis, accompanied by their respective gene names, functions, and potential relevance.

## Data Availability

The proteomic data have been deposited in ProteomeXchange via the PRIDE database with identifier PXD040526.
