# Supplementary material for: Plasma Profiling of Acute Myeloid Leukemia With Fever‐ and Infection‐Related Complications During Chemotherapy‐Induced Neutropenia
Source: Cancer Rep (Hoboken). 2024 Oct 23;7(10):e70024. doi: 10.1002/cnr2.70024 (PMC11498059; doi:10.1002/cnr2.70024)
Supplement: Supplementary file 1 — Figure S1: Study design (A) Patient Cohort: 26 AML patients with chemo‐induced neutropenia underwent longitudinal blood sampling (neutrophil count < 0.5 × 10^9/L). Samples categorized by FN status: non‐fever (triangles), mild‐FN (dots), complicated‐FN (squares). (B) Proteomic Setup and Analysis:Plasma proteins were digested into peptides and analyzed via label‐free LC–MS/MS. Data processed using MaxQuant and Rstudio. (C) Bioinformatic Analysis Flow Chart: MaxQuant analysis produced protein output files. All steps of data analysis corresponded with the figures. Figure S2: Longitudinal protein‐specific coefficients of variation (CVs) calculated per patient (BIOM) vs. the log‐2‐fold difference from first time point of each individual and the average of the subsequent time points. Figure S3: LFQ‐intensities of four proteins that correlated with fever cause from the Spearman correlation analysis. The levels C‐reactive protein (CRP), polymeric immunoglobulin receptor (PIGR), serum amyloid A1 (SAA1), and serum amyloid A2 (SAA2) are plotted across different fever causes, bacterial infection, fungal infection, multifactorial, non‐infectious, unknown focus. [file CNR2-7-e70024-s009.docx]

**Plasma profiling of acute myeloid leukemia with fever- and infection-related complications during chemotherapy-induced neutropenia**

**Supplemental Figures**

Supplemental Figure S1:


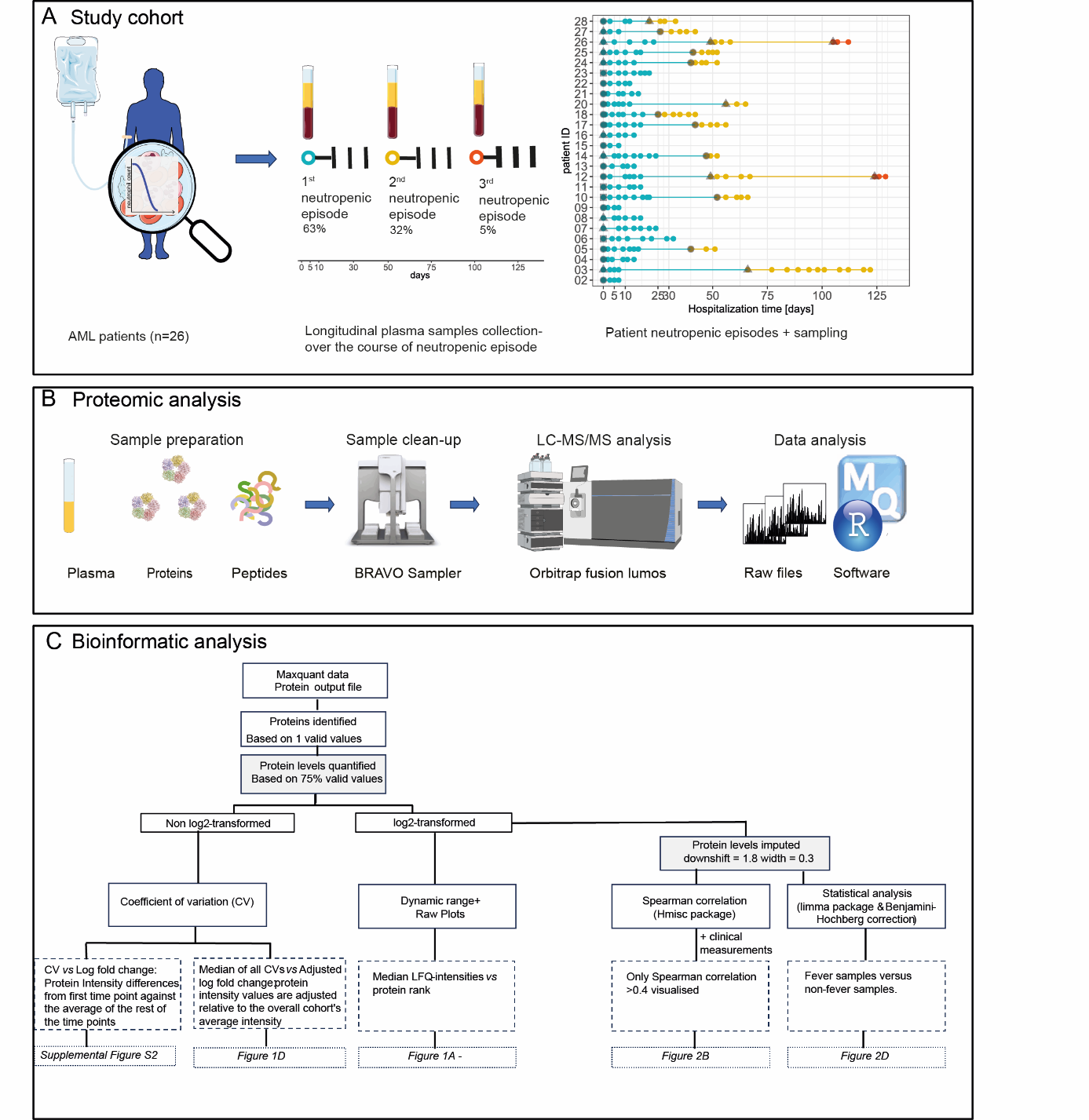


**Supplemental Figure S1: Study design** A) Patient Cohort:26 AML patients with chemo-induced neutropenia underwent longitudinal blood sampling (neutrophil count <0.5 × 10^9/L). Samples categorized by FN status: non-fever (triangles), mild-FN (dots), complicated-FN (squares). B) Proteomic Setup and Analysis:Plasma proteins were digested into peptides and analyzed via label-free LC-MS/MS. Data processed using MaxQuant and Rstudio. C) Bioinformatic Analysis Flow Chart: MaxQuant analysis produced protein output files. All steps of data analysis corresponded with the figures/

**Supplemental Figure S2**


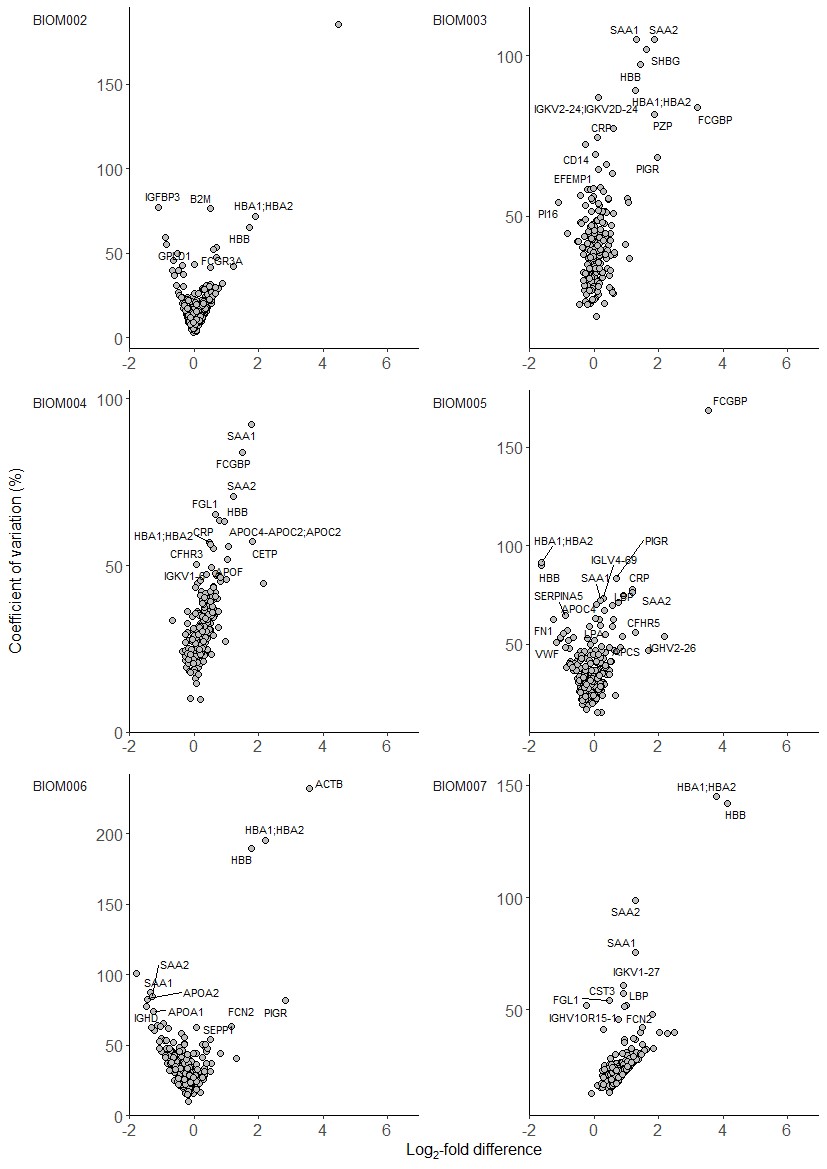


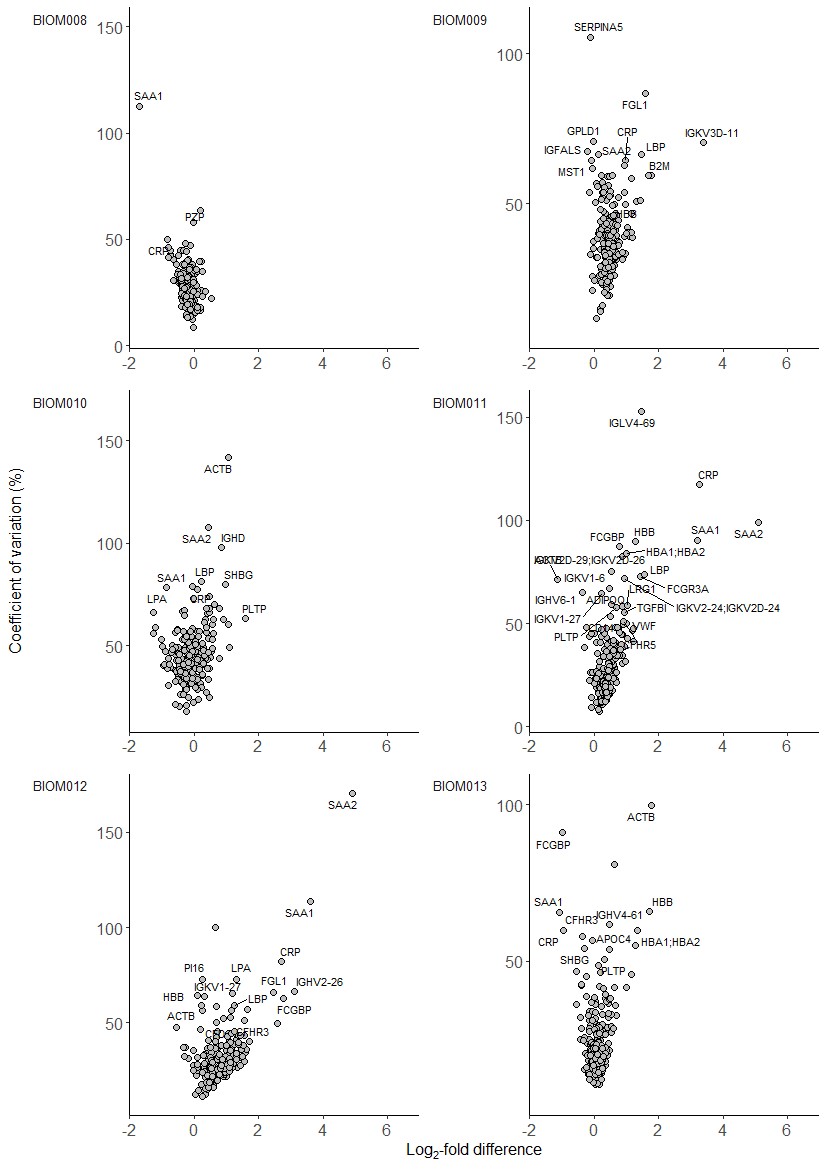


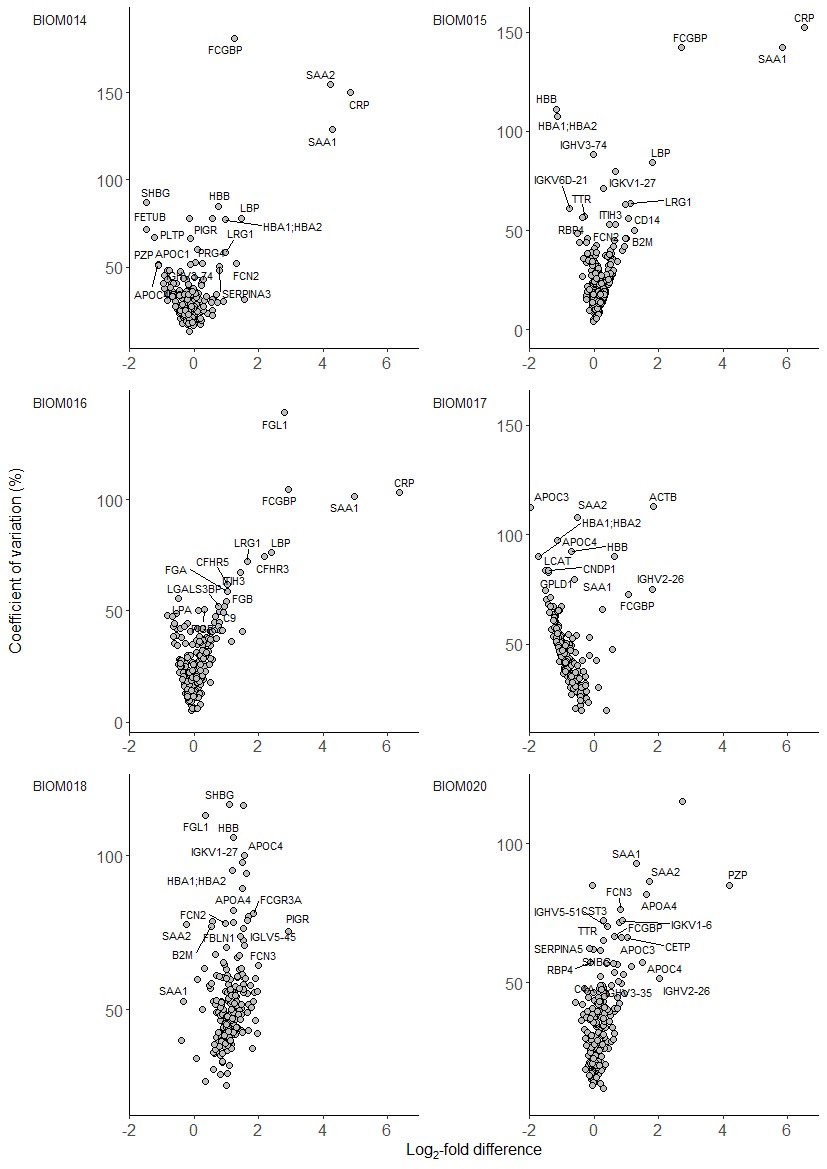


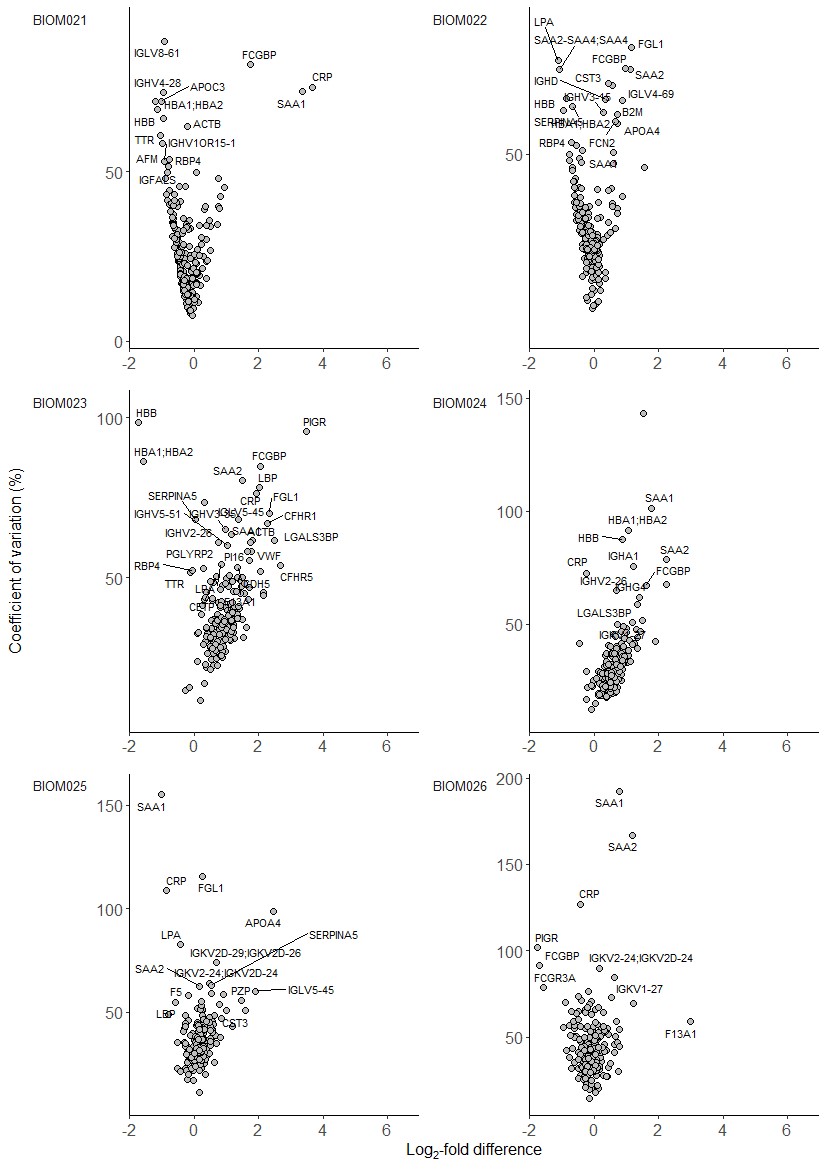


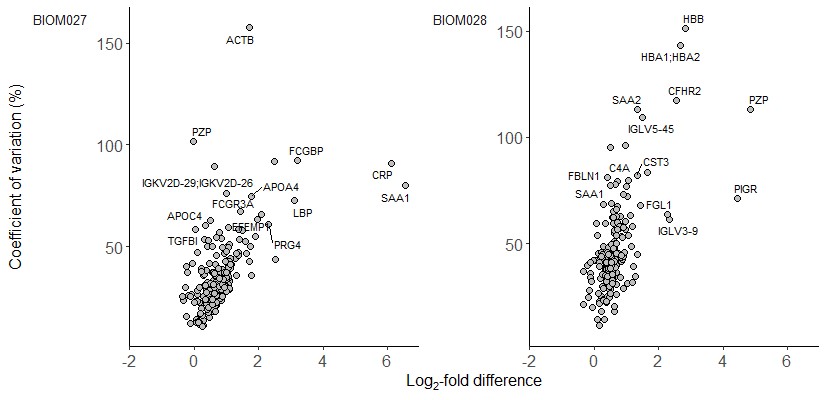


**Supplemental Figure S2:** Longitudinal protein-specific coefficients of variation (CVs) calculated per patient (BIOM) vs the log-2-fold difference from first time point of each individual and the average of the subsequent time points.

**Supplemental Figure S3**


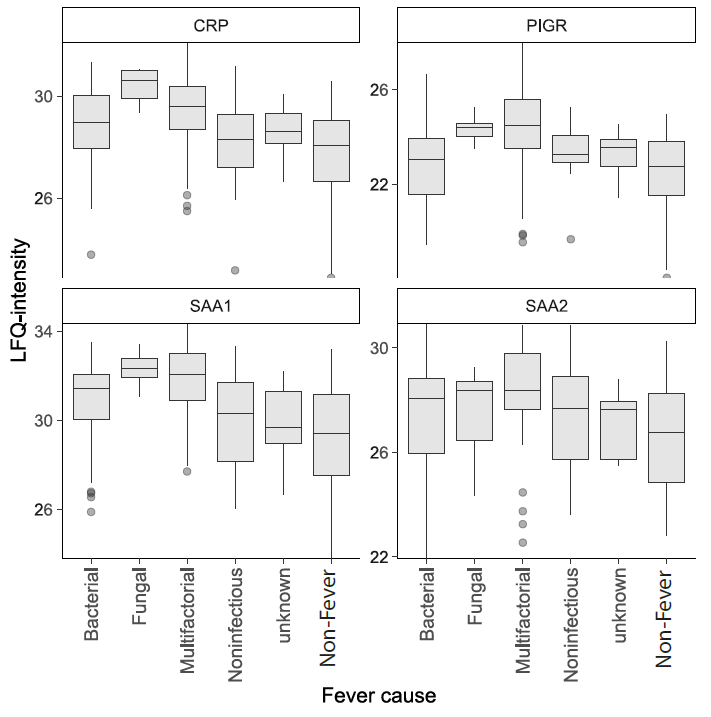


**Supplemental Figure S3:** LFQ-intensities of four proteins that correlated with fever cause from the Spearman correlation analysis. The levels C-reactive protein (CRP), polymeric immunoglobulin receptor (PIGR), serum amyloid A1 (SAA1), and serum amyloid A2 (SAA2) are plotted across different fever causes, bacterial infection, fungal infection, multifactorial, non-infectious, unknown focus.
