# Supplementary material for: Plasma Profiling of Acute Myeloid Leukemia With Fever‐ and Infection‐Related Complications During Chemotherapy‐Induced Neutropenia
Source: Cancer Rep (Hoboken). 2024 Oct 23;7(10):e70024. doi: 10.1002/cnr2.70024 (PMC11498059; doi:10.1002/cnr2.70024)
Supplement: Supplementary file 2 — Table S1: Patient characteristics and details on the febrile neutropenic episodes adapted from van de Geer et al. [6]. [file CNR2-7-e70024-s001.docx]

**Table S1: Patient characteristics and details on the febrile neutropenic episodes adapted from van de Geer et al. [6].**

| Characteristic | number |
| --- | --- |
| Patients, n | 26 |
| Male, n (%) | 17 (65.4) |
| Age at inclusion, years, median (IQR) | 59.5 [46.7–64.9] |
| Male | 59.8 [49–66.6] |
| Female | 48.5 [43.8–63.6] |
| *AML type* at study inclusion, n/N (%) |  |
| Relapse | 4/26 (15.4) |
| Good Risk | 7/26 (26.9) |
| Poor Risk | 8/26 (30.8) |
| Very Poor Risk | 2/26 (7.7) |
| Intermediate Risk | 2/26 (7.7) |
| Good risk Acute Promyelocytic Leukemia | 1/26 (3.8) |
| Acute Myelo-monocytic Leukemia, intermediate/poor risk | 1/26 (3.8) |
| Acute Myelo-monocytic Leukemia, intermediate risk | 1/26 (3.8) |
| Duration of sampling, days, median [IQR] | 32.5 [14.5- 52.0] |
| Duration of neutropenia, days, median [IQR] | 15 [12.3–21.3] |
| Neutropenic episodes, n | 40 |
| Patients with 1 neutropenic episode, n (%) | 13/26 (50) |
| Patients with 2 neutropenic episodes, n (%) | 12/26 (46) |
| Patients with 3 neutropenic episodes, n (%) | 1/26 (4) |
| Patients with febrile neutropenia, n | 21 |
| Febrile neutropenic episodes, n | 26 |
| Patients with 1 neutropenic episode, n (%) | 13/26 (50) |
| Patients with 2 neutropenic episodes, n (%) | 11/26 (42) |
| Patients with 3 neutropenic episodes, n (%) | 2/26 (8) |
| Days until fever onset, median ([QR] | 5.5 [1.0–8.5] |
| Cause of fever, *n/N* (%) |  |
| Bacterial infection | 9/26 (34.6) |
| Viral infection | 0/26 |
| Fungal infection | 2/26 (7.7) |
| Multifactorial | 8/26 (30.8) |
| Non-infectious | 4/26 (15.4) |
| Unknown focus | 3/26 (11.5) |
| Patients with confirmed bloodstream infection, *n/N* (%) | 12/26 (46) |
| Infection-related ICU admission, *n/N* (%) | 4/26 (15) |
| Infection-related 28-day mortality, *n/N* (%) | 3/26 (11.5) |
