# Supplementary material for: Plasma Profiling of Acute Myeloid Leukemia With Fever‐ and Infection‐Related Complications During Chemotherapy‐Induced Neutropenia
Source: Cancer Rep (Hoboken). 2024 Oct 23;7(10):e70024. doi: 10.1002/cnr2.70024 (PMC11498059; doi:10.1002/cnr2.70024)
Supplement: Supplementary file 3 — Table S2: Characteristics of patients, including the total number of samples analyzed, the neutropenic episodes experienced, and the number of samples per neutropenic episode. [file CNR2-7-e70024-s005.docx]

**Supplemental Table S2: Characteristics of patients, including the total number of samples analyzed, the neutropenic episodes experienced, and the number of samples per neutropenic episode.**

| **Patient**  **identifier** | **Neutropenic episode** | **Samples per neutropenic episode** |
| --- | --- | --- |
| BIOM002 | 1 | 4 |
| BIOM003 | 1 | 4 |
|  | 2 | 11 |
| BIOM004 | 1 | 6 |
| BIOM005 | 1 | 7 |
|  | 2 | 3 |
| BIOM006 | 1 | 8 |
| BIOM007 | 1 | 7 |
| BIOM008 | 1 | 6 |
| BIOM009 | 1 | 4 |
| BIOM010 | 1 | 8 |
|  | 2 | 5 |
| BIOM011 | 1 |  |
| BIOM012 | 1 | 6 |
|  | 2 | 5 |
|  | 3 | 3 |
| BIOM013 | 1 | 5 |
| BIOM014 | 1 | 8 |
|  | 2 | 3 |
| BIOM015 | 1 | 3 |
| BIOM016 | 1 | 5 |
| BIOM017 | 1 | 7 |
|  | 2 | 5 |
| BIOM018 | 1 | 7 |
|  | 2 | 6 |
| BIOM020 | 1 | 6 |
|  | 2 | 3 |
| BIOM021 | 1 | 6 |
| BIOM022 | 1 | 5 |
| BIOM023 | 1 | 8 |
| BIOM024 | 1 | 5 |
|  | 2 | 5 |
| BIOM025 | 1 | 6 |
|  | 2 | 5 |
| BIOM026 | 1 | 5 |
|  | 2 | 4 |
|  | 3 | 3 |
| BIOM027 | 1 | 3 |
|  | 2 | 5 |
| BIOM028 | 1 | 4 |
|  | 2 | 4 |
