# Supplementary material for: Plasma Profiling of Acute Myeloid Leukemia With Fever‐ and Infection‐Related Complications During Chemotherapy‐Induced Neutropenia
Source: Cancer Rep (Hoboken). 2024 Oct 23;7(10):e70024. doi: 10.1002/cnr2.70024 (PMC11498059; doi:10.1002/cnr2.70024)
Supplement: Supplementary file 4 — Table S3: Overview of Study Cohort and Analytical Procedures Described in the Methods Section. [file CNR2-7-e70024-s010.docx]

**Table S3: Overview of Study Cohort and Analytical Procedures Described in the Methods Section.**

| **Header** | **Name** | **Description** |
| --- | --- | --- |
| Study cohort | Study cohort | A group of patients involved in a study, including details such as number of participants, diagnoses, treatment regimens, and risk categories. |
|  | Neutropenic episodes | Episodes categorized into non-FN, mild-FN, and complicated-FN. |
|  | Non-FN | Episodes without fever. |
|  | mild-FN | Episodes of febrile neutropenia. |
|  | complicated-FN | Episodes of fever with infection-related complications |
|  |  |  |
|  |  |  |
| Blood collection and conventional laboratory tests | Blood collection | Blood obtained by venipuncture using the vacutainer system. Plasma obtained by centrifugation and stored at -80°C until analysis. |
|  | ELISA | Enzyme-Linked Immunosorbent Assay |
|  | CRP | C-reactive protein |
|  | MRP8/14 | myeloid-related protein-8/14 |
|  | IL6 | Interleukin-6 |
|  | HNE | human neutrophil elastase |
|  |  |  |
|  |  |  |
| Sample preparation for proteomic analysis | Sample preparation | Steps to prepare blood samples for proteomic analysis, including dilution, incubation, digestion, and desalting procedures. |
|  |  |  |
|  | AssayMAP Bravo platform | Platform used for automated solid phase extraction (SPE) of peptides before LC-MS/MS analysis. |
|  |  |  |
|  |  |  |
| Proteomic analysis | Proteomic analysis | Process of analyzing the proteome of the samples, involving LC-MS/MS, data acquisition settings, and specific equipment used. |
|  | Mass spectrometry (MS) | Mass spectrometer |
|  | Tandem MS (MS/MS) | Tandem mass spectrometry |
|  | Liquid chromatography (LC) | Liquid chromatography |
|  | Buffer A | Buffer composed of 0.1% formic acid used in LC-MS/MS analysis. |
|  | LC-MS run type | Type of LC-MS run, usually 'Standard' for conventional shotgun proteomics run with data-dependent MS/MS. |
|  | C18 column | Column with octadecyl carbon chain (C18) bonded silica particles, commonly used in reversed-phase liquid chromatography. |
|  | Survey scans | Scans of peptide precursors from 375 to 1500 m/z performed at target. |
|  | HCD fragmentation | High-energy collision dissociation used for fragmenting peptides in mass spectrometry. |
|  |  |  |
|  |  |  |
| Data analysis | Data analysis | Steps for processing RAW files with MaxQuant software, including searching against the Homo Sapiens Uniprot database, filtering proteins, and handling missing values through imputation. |
|  | RAW files | Files acquired from Xcalibur software and processed with MaxQuant (MQ) 1.6.2.10 software. |
|  | MaxQuant software (MQ) | Software used to analyze proteomics data. |
|  | Imputation | Missing values imputed by drawing random samples from a normal distribution with a mean shifted downward by 1.8 standard deviation and scaled to 0.3 relative to the protein abundance. |
|  | Protein.IDs | Identified proteins from mass spectrometry data |
|  | UID | Unique identifiers for proteins |
|  | LFQ | Label-free quantitation |
|  |  |  |
|  |  |  |
| Bioinformatics analysis | Bioinformatics analysis | Analysis of protein variation, correlation with clinical measurements, network construction, and statistical analysis. |
|  | Coefficient of variation (CV) | Calculation of protein variation within and across patients. |
|  | Log-fold change (LFC) | Adjusted log-fold change to normalize protein intensity values across all samples. |
|  | Spearman correlation | Calculation of correlation between quantified proteins and clinical measurements. |
|  | Benjamin Hochberg (BH) |  |
|  |  |  |
|  |  |  |
| Data availability | Data availability | Availability of raw MS files and search/identification files in the ProteomeXchange consortium via the PRIDE partner repository with identifier PXD040526. |
|  | PRIDE | PRoteomics IDEntifications (PRIDE) database for proteomics data submission and retrieval. |
