# Supplementary material for: Plasma Profiling of Acute Myeloid Leukemia With Fever‐ and Infection‐Related Complications During Chemotherapy‐Induced Neutropenia
Source: Cancer Rep (Hoboken). 2024 Oct 23;7(10):e70024. doi: 10.1002/cnr2.70024 (PMC11498059; doi:10.1002/cnr2.70024)
Supplement: Supplementary file 10 — Table S9: A list of proteins that exhibit correlations with clinical and laboratory data in correlation analysis, accompanied by their respective gene names, functions, and potential relevance. [file CNR2-7-e70024-s004.docx]

**Supplemental Table S9:** **A list of proteins that exhibit correlations with clinical and laboratory data in correlation analysis, accompanied by their respective gene names, functions, and potential relevance.**

| Gene name | protein name | UniProt identifier | Function description uniport | Potential relevance | |
| --- | --- | --- | --- | --- | --- |
| ACTB | Actin, cytoplasmic 1 | P60709 | Actin is a highly conserved protein that polymerizes to produce filaments that form cross-linked networks in the cytoplasm of cells |  | |
| CD14 | Monocyte differentiation antigen CD14 | P08571 | Coreceptor for bacterial lipopolysaccharide. In concert with LBP, binds to monomeric lipopolysaccharide and delivers it to the LY96/TLR4 complex, thereby mediating the innate immune response to bacterial lipopolysaccharide (LPS) | Presepsin, also referred as soluble CD14 subtype, is associated with inflammation (Ahmed et al., 2021) |  |
| CRP | C-reactive protein | Q07203 | Displays several functions associated with host defence: it promotes agglutination, bacterial capsular swelling, phagocytosis, and complement fixation through its calcium-dependent binding to phosphorylcholine. | CRP is known as a common marker for inflammation (Anush et al., 2019; Geyer, Kulak, et al., 2016) | |
| FCGBP | IgGFc-binding protein | Q9Y6R7 | May be involved in the maintenance of the mucosal structure as a gel-like component of the mucosa. |  |  |
| FGL1 | Fibrinogen-like protein 1 | Q08830 | Immune suppressive molecule that inhibits antigen-specific T-cell activation by acting as a major ligand of LAG3. | FGL1 functions in regulating immune response and has been suggested as a therapeutic target in inflammatory conditions.(Sui et al., 2022) | |
| ITIH3 | Inter-alpha-trypsin inhibitor heavy chain H3 | Q06033 | May act as a carrier of hyaluronan in serum or as a binding protein between hyaluronan and other matrix protein, including those on cell surfaces in tissues to regulate the localization, synthesis and degradation of hyaluronan which are essential to cells undergoing biological processes. |  |  |
| LBP | Lipopolysaccharide-binding protein | P18428 | Plays a role in the innate immune response. | LBP have been reported to be elevated in infectious diseases like COVID-19.(Messner et al., 2020; Suvarna et al., 2021) | |
| LGALS3BP | Galectin-3-binding protein | Q08380 | Promotes integrin-mediated cell adhesion. May stimulate host defense against viruses | LGALS3BP has been previously described to have both prognostic and functional roles in cancer (Capone et al., 2021) |  |
| LRG1 | Leucine-rich alpha-2-glycoprotein 1 | P02750 | plays a crucial role in the proliferation and apoptosis of colorectal cancer (CRC) by regulating RUNX1 expression. | LRG1, has recently emerged as a potential biomarker in cancer patients at high risk for disease progression and recurrence.(Hoefsmit et al., 2023) | |
| PIGR | Polymeric immunoglobulin receptor | P01833 | Mediates selective transcytosis of polymeric IgA and IgM across mucosal epithelial cells. Binds polymeric IgA and IgM at the basolateral surface of epithelial cells. The complex is then transported across the cell to be secreted at the apical surface. During this process, a cleavage occurs that separates the extracellular (known as the secretory component) from the transmembrane segment. | PIGR have been reported to be elevated in infectious diseases like COVID-19.(Messner et al., 2020; Suvarna et al., 2021) |  |
| PLTP | Phospholipid transfer protein | P55058 | Mediates the transfer of phospholipids and free cholesterol from triglyceride-rich lipoproteins (low density lipoproteins or LDL and very low density lipoproteins or VLDL) into high-density lipoproteins (HDL) as well as the exchange of phospholipids between triglyceride-rich lipoproteins themselves |  | |
| SAA1 | Serum amyloid A-1 protein | P0DJI8 | Major acute phase protein. | SAA1 is known as a common marker for inflammation (Anush et al., 2019; Geyer, Kulak, et al., 2016) | |
| SAA2 | Serum amyloid A-2 protein | P0DJI9 | Major acute phase reactant. | SAA2 is known as a common marker for inflammation (Anush et al., 2019; Geyer, Kulak, et al., 2016) |  |
| SERPINA3 | Serpin family A member 3 | P01011 | Although its physiological function is unclear, it can inhibit neutrophil cathepsin G and mast cell chymase, both of which can convert angiotensin-1 to the active angiotensin-2 |  | |
